# Supplementary material for: Is School Gardening Combined with Physical Activity Intervention Effective for Improving Childhood Obesity? A Systematic Review and Meta-Analysis
Source: Nutrients. 2021 Jul 28;13(8):2605. doi: 10.3390/nu13082605 (PMC8402215; doi:10.3390/nu13082605)
Supplement: Supplementary file 1 [file nutrients-13-02605-s001.zip › supplementary 2.pdf]

Sensitivity Analysis Outcomes (Figure S1), and the source of heterogeneity is Davis' study

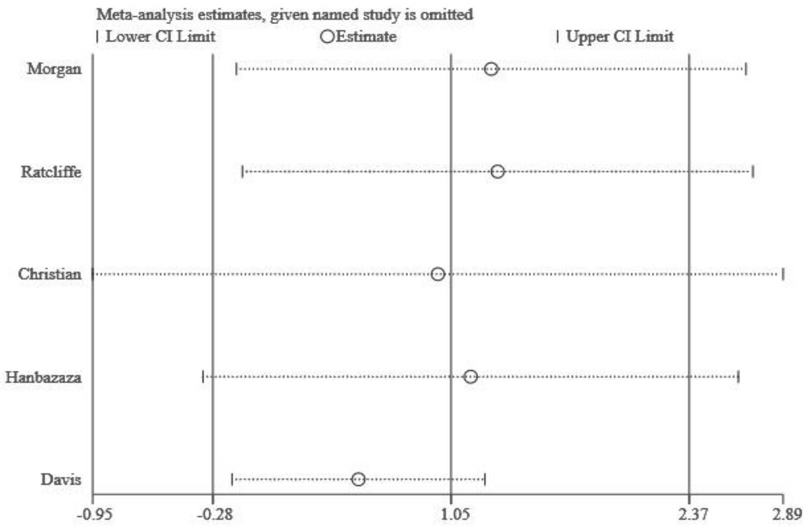

Figure S1. Sensitivity analysis of FVs combined results

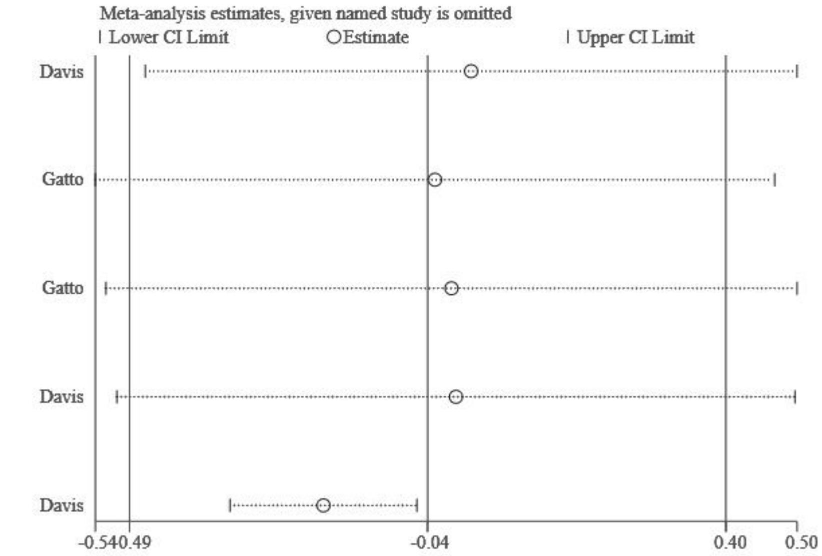

Figure S2: Sensitivity analysis of BMI combined results

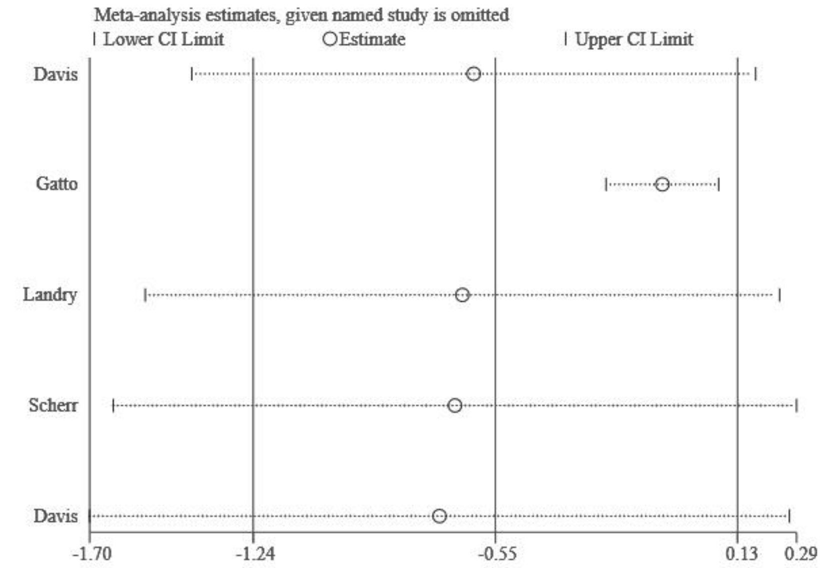

Figure S3: Sensitivity analysis of BMI z-score combined results

We carried out sensitivity analysis on the 6 included studies, and the results showed that Gatto's study was the source of heterogeneity in WC combined results (Figure S4).

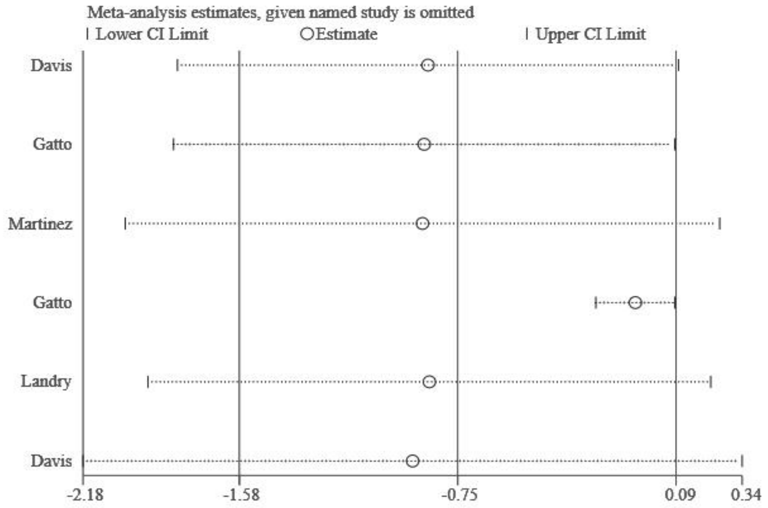

Figure S4: Sensitivity analysis of WC combined results

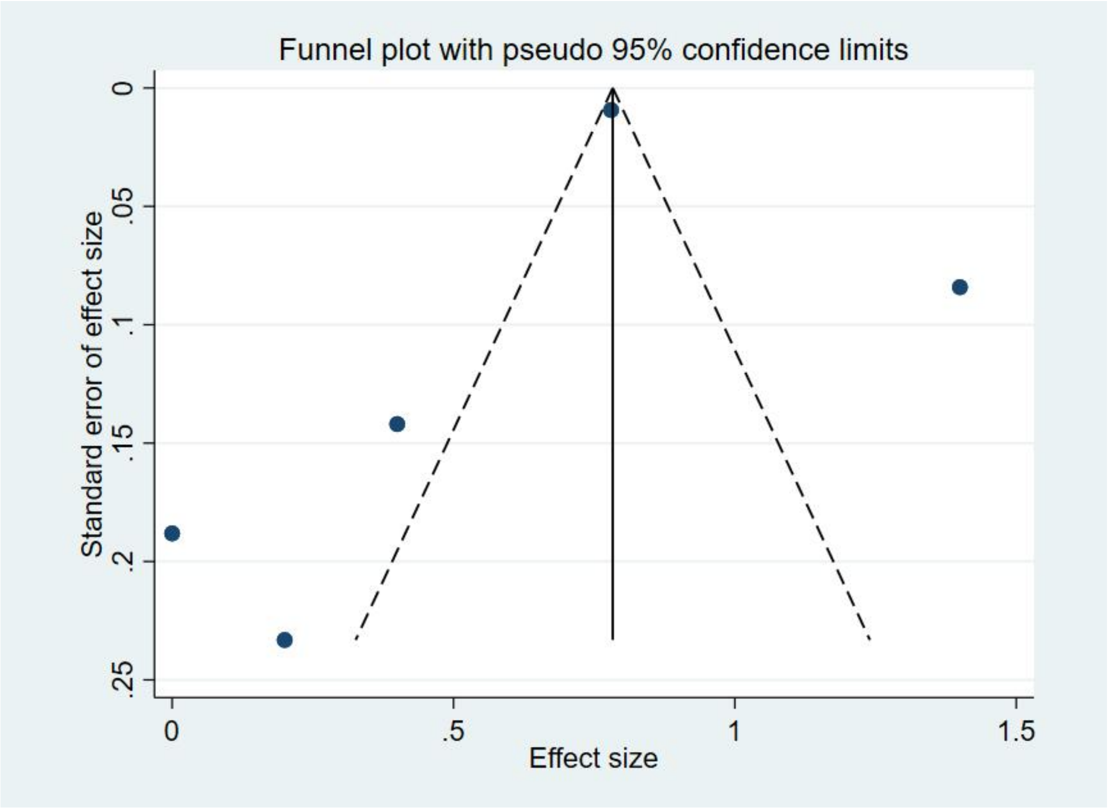

Figure S5: funnel plot of FVs

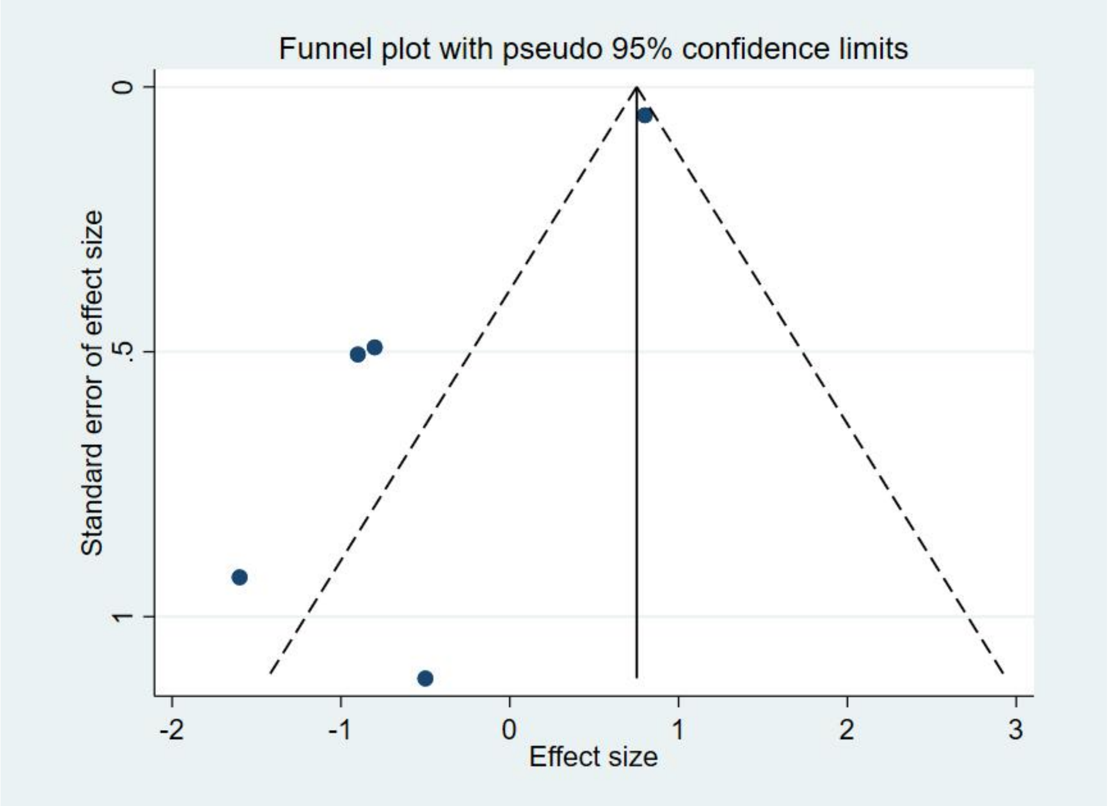

.

Figure S6: funnel plot of BMI

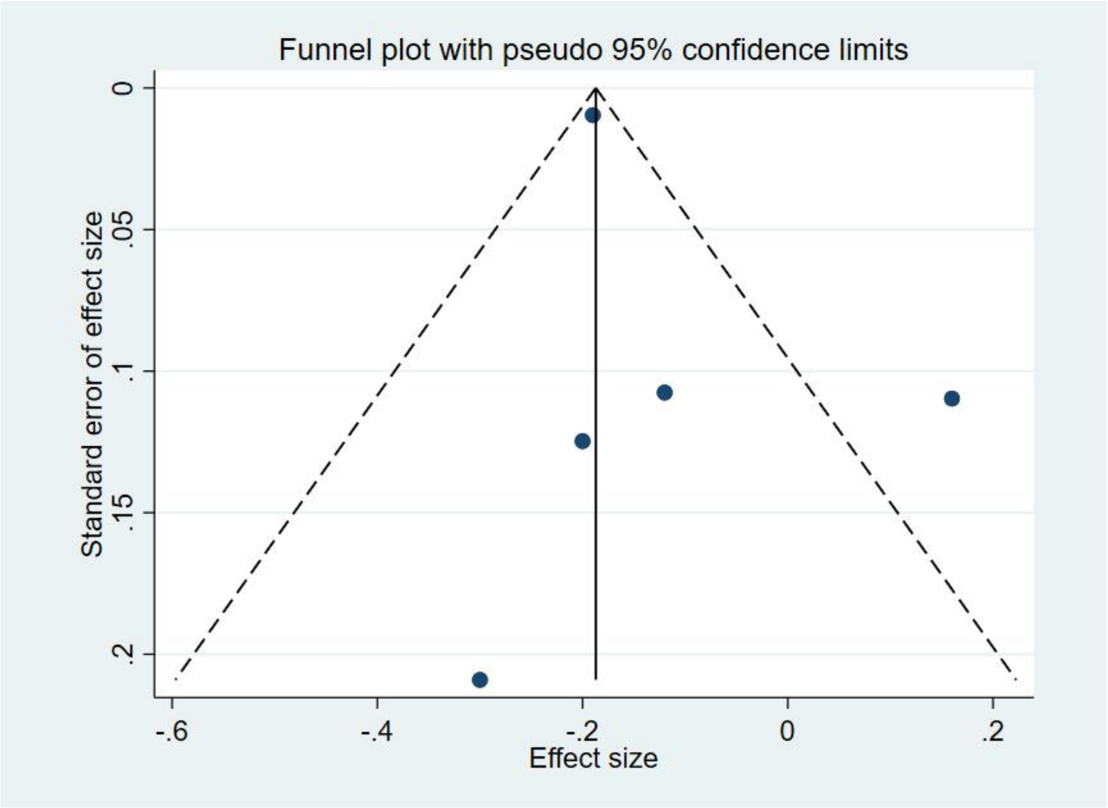

Figure S7: funnel plot of BMI-z

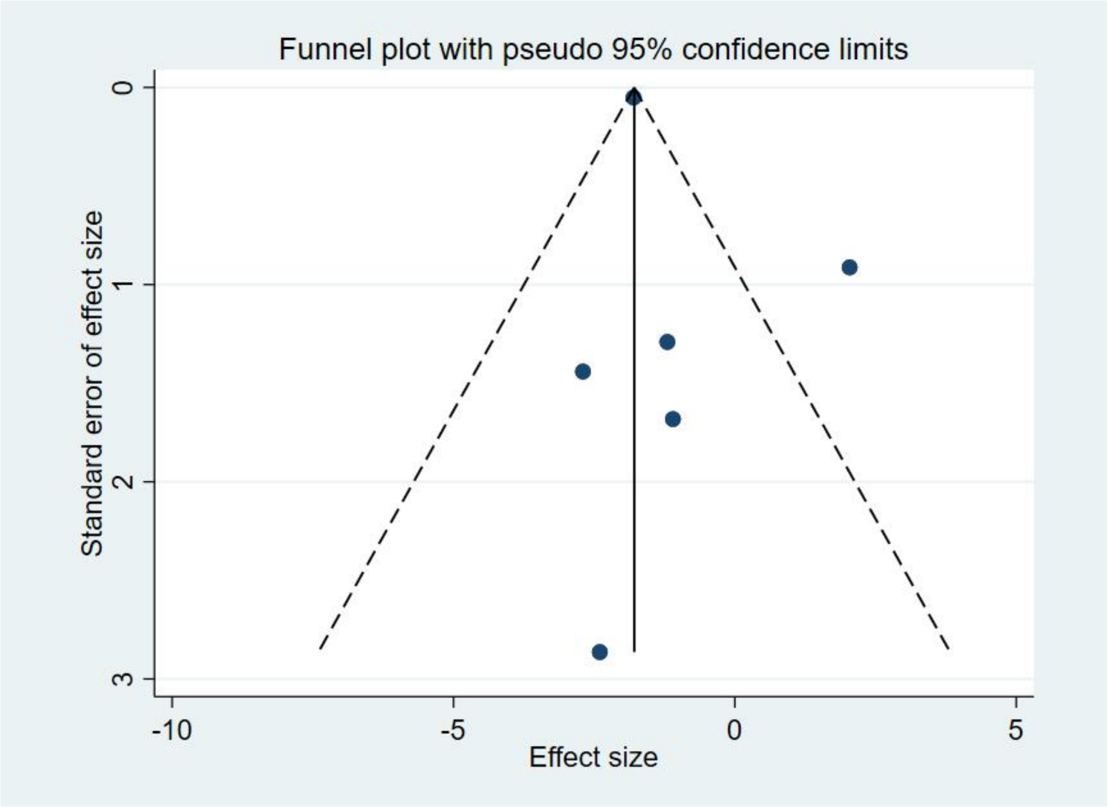

Figure S8: funnel plot of WC

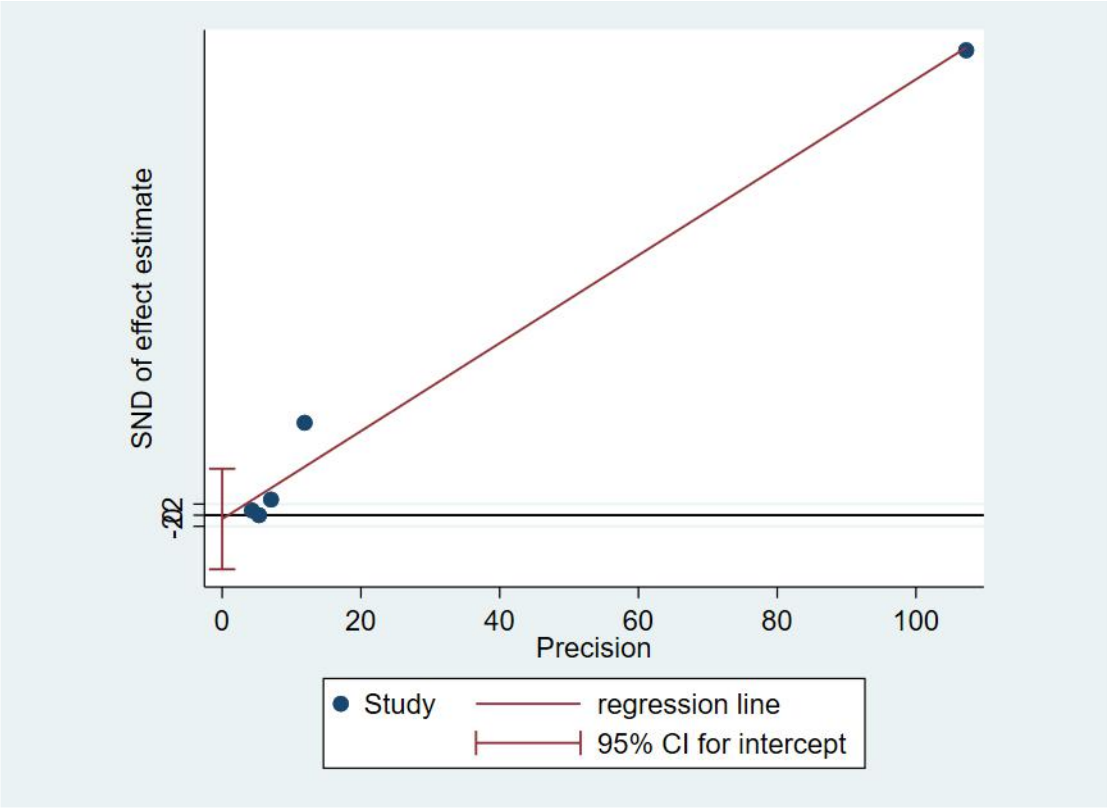

Figure S9: Egger’s test of FVs

Egger's test for small-study effects:
Regress standard normal deviate of intervention
effect estimate against its standard error

Number of studies = **5** Root MSE = **5.263**

Std_Eff	Coef.	Std. Err.	t	P>|t|	[95% Conf.	Interval]
slope	**.7910224**	**.0586582**	**13.49**	**0.001**	**.6043457**	**.9776991**
bias	**-.6876996**	**2.842081**	**-0.24**	**0.824**	**-9.732471**	**8.357072**

Test of H0: no small-study effects P = **0.824**

Figure S10: Egger’s test’s data of FVs

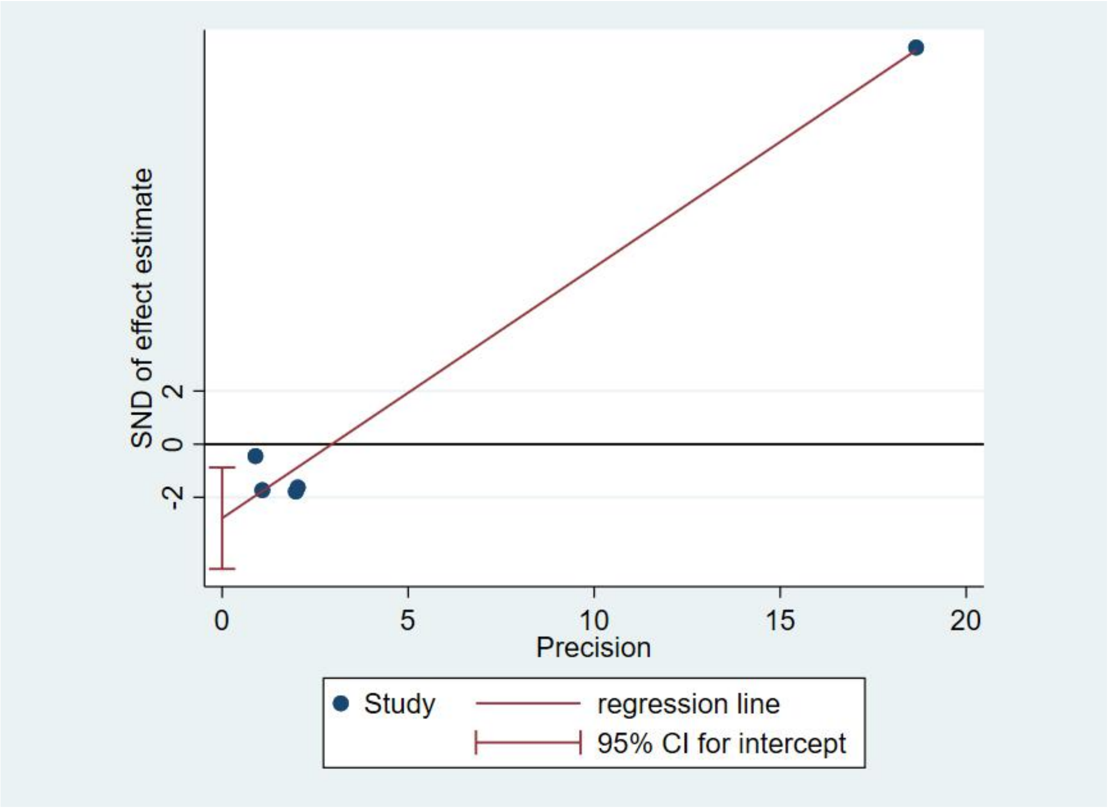

Figure S11: Egger's test of BMI

Egger's test for small-study effects:
Regress standard normal deviate of intervention
effect estimate against its standard error

Number of studies = **5** Root MSE = **1.091**

Std_Eff	Coef.	Std. Err.	t	P>|t|	[95% Conf.	Interval]
slope	**.9438275**	**.0709091**	**13.31**	**0.001**	**.7181632**	**1.169492**
bias	**-2.784353**	**.5999996**	**-4.64**	**0.019**	**-4.69382**	**-.8748864**

Test of H0: no small-study effects P = **0.019**

Figure S12: Egger's test's data of BMI

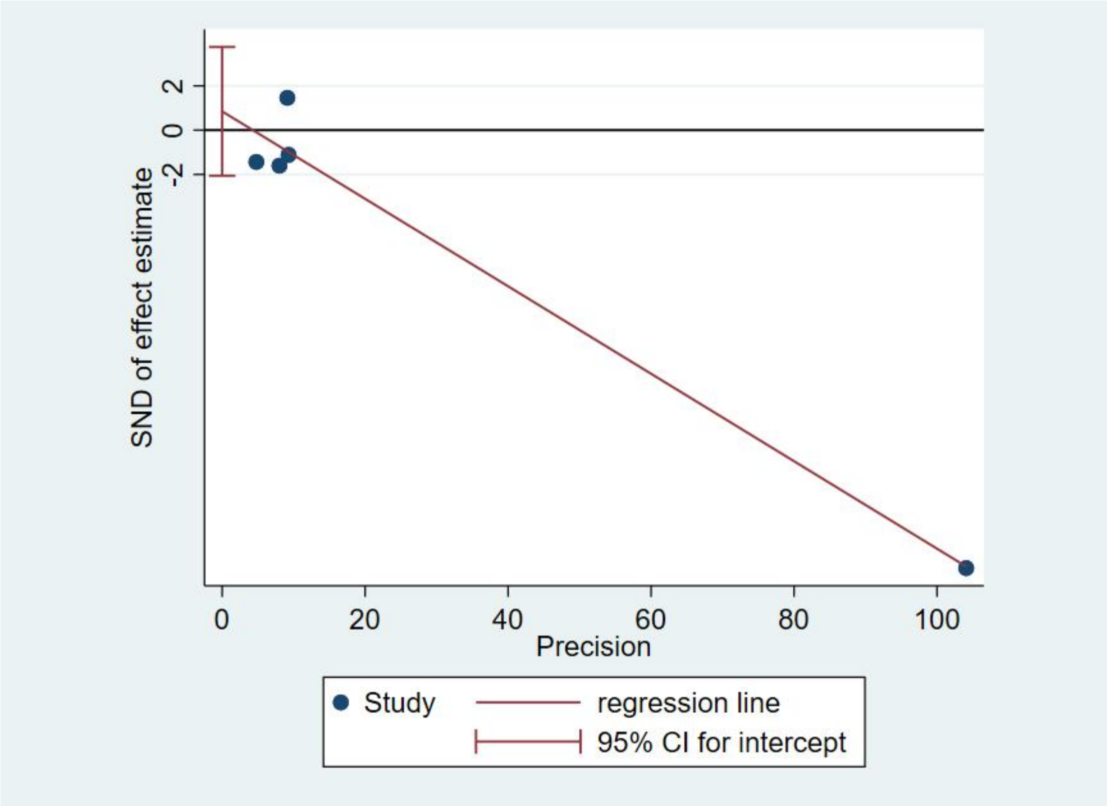

Figure S13: Egger’s test of BMI-z

```
Egger's test for small-study effects:
Regress standard normal deviate of intervention
effect estimate against its standard error

Number of studies =   5                            Root MSE      =    1.671
------------------------------------------------------------------------------
     Std_Eff |      Coef.   Std. Err.       t    P>|t|     [95% Conf. Interval]
-------------+----------------------------------------------------------------
       slope |  -.1974798   .0193923   -10.18    0.002    -.2591949   -.1357647
        bias |   .8494904   .9132788     0.93    0.421     -2.05697    3.755951
------------------------------------------------------------------------------

Test of H0: no small-study effects          P = 0.421
```

Figure S14: Egger’s test’s data of BMI-z

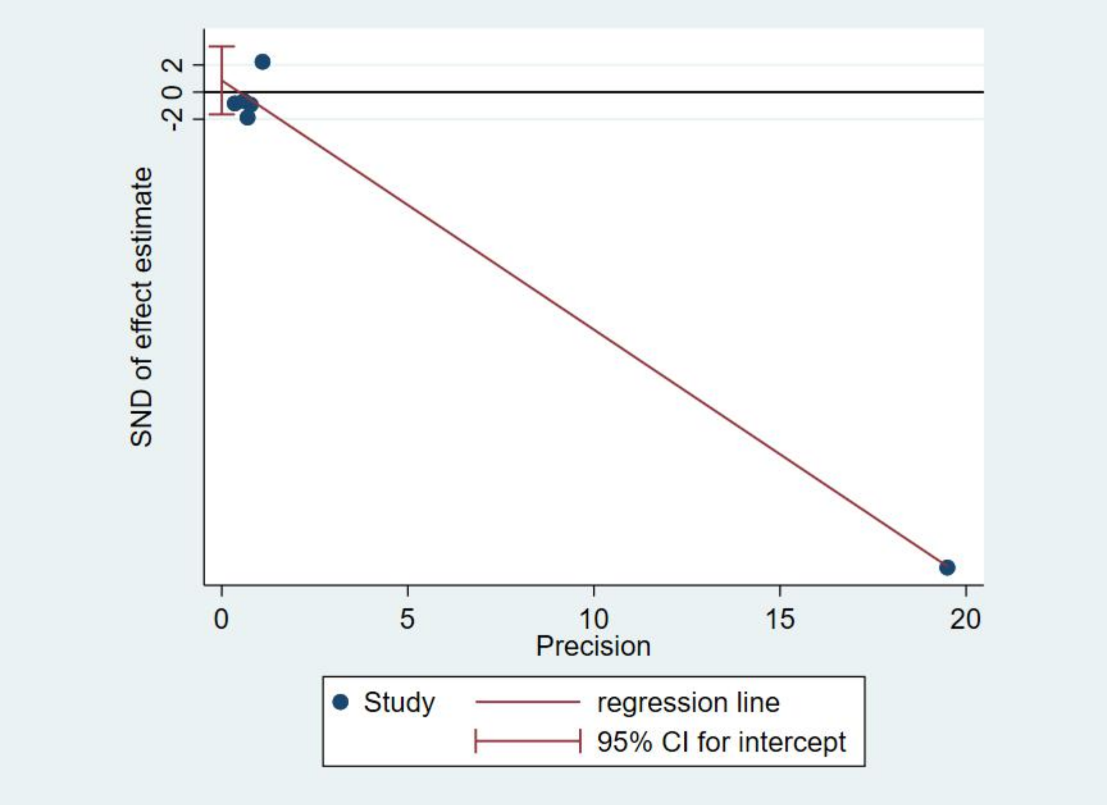

Figure S15: Egger's test's of WC

Egger's test for small-study effects:
Regress standard normal deviate of intervention
effect estimate against its standard error

Number of studies = **5** Root MSE = **1.091**

Std_Eff	Coef.	Std. Err.	t	P>|t|	[95% Conf.	Interval]
slope	**.9438275**	**.0709091**	**13.31**	**0.001**	**.7181632**	**1.169492**
bias	**-2.784353**	**.5999996**	**-4.64**	**0.019**	**-4.69382**	**-.8748864**

Test of H0: no small-study effects P = **0.019**

Figure S16: Egger's test's data of WC
