# Supplementary material for: Is School Gardening Combined with Physical Activity Intervention Effective for Improving Childhood Obesity? A Systematic Review and Meta-Analysis
Source: Nutrients. 2021 Jul 28;13(8):2605. doi: 10.3390/nu13082605 (PMC8402215; doi:10.3390/nu13082605)
Supplement: Supplementary file 1 [file nutrients-13-02605-s001.zip › Supplementary 3.pdf]

Table S2. Search strategy (example for 2 databases).

Pubmed	Search terms for query	Results
#1	(((((((((((((((((Exercise[MeSH Terms]) OR (Exercises[MeSH Terms])) OR (Physical Activity[MeSH Terms])) OR (Activities, Physical[MeSH Terms])) OR (Exercise, Physical[MeSH Terms])) OR (Exercises, Physical[MeSH Terms])) OR (Acute Exercise[MeSH Terms])) OR (Acute Exercises[MeSH Terms])) OR (Exercise, Isometric[MeSH Terms])) OR (Exercises, Isometric[MeSH Terms])) OR (Exercise, Aerobic[MeSH Terms])) OR (Aerobic Exercises[MeSH Terms])) OR (Exercise Training[MeSH Terms])) OR (Exercise Trainings[MeSH Terms])) OR (Gardening[MeSH Terms])) OR (Horticultural Therapy[MeSH Terms])) OR (Gardening Therapy[MeSH Terms])) OR (Therapeutic Horticulture[MeSH Terms])) OR (Horticulture Therapy[MeSH Terms])) OR (Garden Therapy[MeSH Terms]) Filters: from 1945/1/1 - 2021/1/31	24522
#2	(((((((((((Pediatric Obesity[MeSH Terms]) OR (Childhood Onset Obesity[MeSH Terms])) OR (Obesity in	387

	Childhood[MeSH Terms])) OR (Child Obesity[MeSH Terms])) OR (Childhood Obesity[MeSH Terms])) OR (Infant Overweight[MeSH Terms])) OR (Infantile Obesity[MeSH Terms])) OR (Infant Obesity[MeSH Terms])) OR (Childhood Overweight[MeSH Terms])) OR (Adolescent Overweight[MeSH Terms])) OR (Adolescent Obesity[MeSH Terms])) OR (Obesity in Adolescence[MeSH Terms])) AND (fruits, vegetables) Filters: from 1945/1/1 - 2021/1/31	
#3	((((((((((((((((((Exercise[MeSH Terms]) OR (Exercises[MeSH Terms])) OR (Physical Activity[MeSH Terms])) OR (Activities, Physical[MeSH Terms])) OR (Exercise, Physical[MeSH Terms])) OR (Exercises, Physical[MeSH Terms])) OR (Acute Exercise[MeSH Terms])) OR (Acute Exercises[MeSH Terms])) OR (Exercise, Isometric[MeSH Terms])) OR (Exercises, Isometric[MeSH Terms])) OR (Exercise, Aerobic[MeSH Terms])) OR (Aerobic Exercises[MeSH Terms])) OR (Exercise Training[MeSH Terms])) OR (Exercise Trainings[MeSH Terms])) OR (Gardening[MeSH Terms])) OR (Horticultural Therapy[MeSH Terms])) OR (Gardening	125

	Therapy[MeSH Terms])) OR (Therapeutic Horticulture[MeSH Terms])) OR (Horticulture Therapy[MeSH Terms])) OR (Garden Therapy[MeSH Terms])) AND ((((((((((((Pediatric Obesity[MeSH Terms]) OR (Childhood Onset Obesity[MeSH Terms])) OR (Obesity in Childhood[MeSH Terms])) OR (Child Obesity[MeSH Terms])) OR (Childhood Obesity[MeSH Terms])) OR (Infant Overweight[MeSH Terms])) OR (Infantile Obesity[MeSH Terms])) OR (Infant Obesity[MeSH Terms])) OR (Childhood Overweight[MeSH Terms])) OR (Adolescent Overweight[MeSH Terms])) OR (Adolescent Obesity[MeSH Terms])) OR (Obesity in Adolescence[MeSH Terms])) AND (fruits,vegetables)) Filters: from 1945/1/1 - 2021/1/31	
#4	(child[MeSH Terms]) OR (children[MeSH Terms]) Filters: from 1945/1/1 - 2021/1/31	1979019
#5	(((((((((((((((((((Exercise[MeSH Terms]) OR (Exercises[MeSH Terms])) OR (Physical Activity[MeSH Terms])) OR (Activities, Physical[MeSH Terms])) OR (Exercise, Physical[MeSH Terms])) OR (Exercises,	103

Physical[MeSH Terms])) OR (Acute Exercise[MeSH Terms])) OR (Acute Exercises[MeSH Terms])) OR (Exercise, Isometric[MeSH Terms])) OR (Exercises, Isometric[MeSH Terms])) OR (Exercise, Aerobic[MeSH Terms])) OR (Aerobic Exercises[MeSH Terms])) OR (Exercise Training[MeSH Terms])) OR (Exercise Trainings[MeSH Terms])) OR (Gardening[MeSH Terms])) OR (Horticultural Therapy[MeSH Terms])) OR (Gardening Therapy[MeSH Terms])) OR (Therapeutic Horticulture[MeSH Terms])) OR (Horticulture Therapy[MeSH Terms])) OR (Garden Therapy[MeSH Terms])) AND ((((((((((((Pediatric Obesity[MeSH Terms]) OR (Childhood Onset Obesity[MeSH Terms])) OR (Obesity in Childhood[MeSH Terms])) OR (Child Obesity[MeSH Terms])) OR (Childhood Obesity[MeSH Terms])) OR (Infant Overweight[MeSH Terms])) OR (Infantile Obesity[MeSH Terms])) OR (Infant Obesity[MeSH Terms])) OR (Childhood Overweight[MeSH Terms])) OR (Adolescent Overweight[MeSH Terms])) OR (Adolescent Obesity[MeSH Terms])) OR (Obesity in Adolescence[MeSH Terms])) AND

	(fruits,vegetables))) AND ((child[MeSH Terms]) OR (children[MeSH Terms])) Filters: from 1945/1/1 - 2021/1/31	

Web of Science	Search terms for query	Results
#1	TI=(Exercise OR Exercises OR Physical Activity OR Activities, Physical OR Exercise, Physical OR Exercises, Physical OR Acute Exercise OR Acute Exercises OR Exercise, Isometric OR Exercises, Isometric OR Exercise, Aerobic OR Aerobic Exercises OR Exercise Training OR Exercise Trainings OR Gardening OR Horticultural Therapy OR Gardening Therapy OR Therapeutic Horticulture OR Horticulture Therapy OR Garden Therapy) Timespan: 1950-01-01 to 2021-01-31	281467
#2	(TI=(Pediatric Obesity OR Childhood Onset Obesity OR Obesity in Childhood OR Child Obesity OR Childhood obesity OR Infant Overweight OR Infantile Obesity OR Infant Obesity OR Childhood Overweight OR Adolescent Overweight OR	740

	Adolescent Obesity OR Obesity in adolescence))<br>AND TS=(fruits OR vegetables)<br>Timespan: 1950-01-01 to 2021-01-31	
#3	#1 AND #2	50
#4	TS=(child OR children)<br>Timespan: 1950-01-01 to 2021-01-31	3329443
#5	#3 AND #4	47
